# Incidence and Risk of Fatal Vehicle Crashes Among Professional Drivers: A Population-Based Study in Taiwan

**DOI:** 10.3389/fpubh.2022.849547

**Published:** 2022-03-08

**Authors:** Jui-Hsiu Tsai, Ya-Hui Yang, Pei-Shan Ho, Trong-Neng Wu, Yue Leon Guo, Pau-Chung Chen, Hung-Yi Chuang

**Affiliations:** ^1^Ph.D. Program in Environmental and Occupational Medicine, College of Medicine, Kaohsiung Medical University and National Health Research Institutes, Kaohsiung, Taiwan; ^2^Department of Psychiatry, Dalin Tzu Chi Hospital, Buddhist Tzu Chi Medical Foundation, Chia-Yi, Taiwan; ^3^College of Medicine, Tzu Chi University, Hualien City, Taiwan; ^4^Department of Health-Business Administration, Fooyin University, Kaohsiung, Taiwan; ^5^Division of Medical Statistics and Bioinformatics, Department of Medical Research, Kaohsiung Medical University Hospital, Kaohsiung Medical University, Kaohsiung, Taiwan; ^6^Department of Oral Hygiene, College of Dental Medicine, Kaohsiung Medical University, Kaohsiung, Taiwan; ^7^Department of Healthcare Administration, Asia University, Taichung, Taiwan; ^8^Environmental and Occupational Medicine, National Taiwan University College of Medicine and National Taiwan University Hospital, Taipei, Taiwan; ^9^National Institute of Environmental Health Science, National Health Research Institutes, Miaoli, Taiwan; ^10^Department of Public Health, Kaohsiung Medical University, Kaohsiung, Taiwan; ^11^Department of Environmental and Occupational Medicine, Kaohsiung Medical University Hospital, Kaohsiung Medical University, Kaohsiung, Taiwan

**Keywords:** fatal vehicle crash, professional drivers, benzodiazepine, speeding, driver-vehicle-road-environment system

## Abstract

Fatal vehicle crashes (FVCs) are among the leading causes of death worldwide. Professional drivers often drive under dangerous conditions; however, knowledge of the risk factors for FVCs among professional drivers remain scant. We investigated whether professional drivers have a higher risk of FVCs than non-professional drivers and sought to clarify potential risk factors for FVCs among professional drivers. We analyzed nationwide incidence rates of FVCs as preliminary data. Furthermore, by using these data, we created a 1:4 professionals/non-professionals preliminary study to compare with the risk factors between professional and non-professional drivers. In Taiwan, the average crude incidence rate of FVCs for 2003–2016 among professional drivers was 1.09 per 1,000 person-years; professional drivers had a higher percentage of FVCs than non-professional drivers among all motor vehicle crashes. In the 14-year preliminary study with frequency-matched non-professional drivers, the risk of FVCs among professional drivers was significantly associated with a previous history of involvement in motor vehicle crashes (adjustment odds ratio [OR] = 2.157; 95% confidence interval [CI], 1.896–2.453), previous history of benzodiazepine use (adjustment OR = 1.385; 95% CI, 1.215–1.579), and speeding (adjustment OR = 1.009; 95% CI, 1.006–1.013). The findings have value to policymakers seeking to curtail FVCs.

## Introduction

Fatal vehicle crashes (FVCs) are one of the major health and social problems worldwide, causing many deaths and economic losses (1, 2). Globally, more than 1.27 million people die in motor vehicle crashes each year, and 20–50 million people sustain injuries in vehicle crashes. Motor vehicle crash injuries were estimated to be the ninth leading cause of death worldwide in 2004 and are predicted to rise to the fifth leading cause by 2030 (3). The risk of FVCs in all drivers is attributable to a variety of factors, including human factors (e.g., male sex, age [novice or elderly driver], low educational level, physical diseases, alcohol use, illicit or prescription drug use, excessive speeding, distracted driving, driver fatigue, and careless driving), equipment factors (e.g., unsafe vehicle design and air bags), environmental factors (e.g., adverse weather, rural or wet roads, poor lighting, and unsafe road infrastructure), enforcement (e.g., seatbelt use, cell phone use while driving, previous history, of traffic offenses or involvement in motor vehicle crashes, swerving, inadequate traffic laws, and poor law enforcement powers), and medical response (e.g., slow emergency medical response time in remote areas) (3–8).

Road transport is the principal form of transport in most countries and is an important driver of social and economic development. Compared with water, air, and rail transports, road transport has a significantly higher risk of fatal crashes (9). Professional drivers face a challenging work environment due to increasing road travel by non-professional and increased road transport of goods due to economic expansion; thus, the working conditions of professional drivers are dangerous and carry the risk of FVCs (10). To date, few epidemiological studies have been published on FVCs among professional drivers (7, 11–13). In addition, data specifically regarding risk factors for FVCs among professional drivers remain scant.

The present study investigated whether professional drivers have a higher risk of FVCs than non-professional drivers. We analyzed nationwide incidence rates of FVCs for the two populations as preliminary data. Furthermore, by using these data, we created a preliminary study to compare the related characteristics and potential risk factors between professional and non-professional drivers in FVCs. Moreover, we suggest some measures for preventing FVCs among professional drivers.

## Methods

### Data Source and Ethics

This study used seven nationwide databases retrieved from the Road Accident Registry of Injurious Crashes maintained by the National Police Agency, Ministry of the Interior (14, 15); the Taiwan National Health Insurance Research Database (NHIRD) (15, 16); and five Taiwanese population-based administrative registries, namely the management information system of substitution maintenance therapy (Ministry of Health and Welfare) and four information management systems concerning illicit drugs that are maintained by the Ministry of Justice (10). The four information systems concerning illicit drugs were the case management system of the Drug Prevention and Control Center, the criminal records processing system, the drug case prosecution case system, and the punitive administration system for illicit drug users (category 3 or 4 narcotics) (Supplementary Table 3). Preliminary data were independently collected by the aforementioned governmental departments and managed by the Health and Welfare Data Science Center, Ministry of Health and Welfare. All data from the various systems were linked using the unique national identification numbers assigned to each resident in Taiwan. Personal identifiers were removed after linking and before the analysis. The study was approved by the relevant institutional review boards (TSMH IRB No.: 17-010-B1 and 18-049-B), and written informed consent was waived because the data we obtained were de-identified.

### Study Population and Design

**Figure 1:**
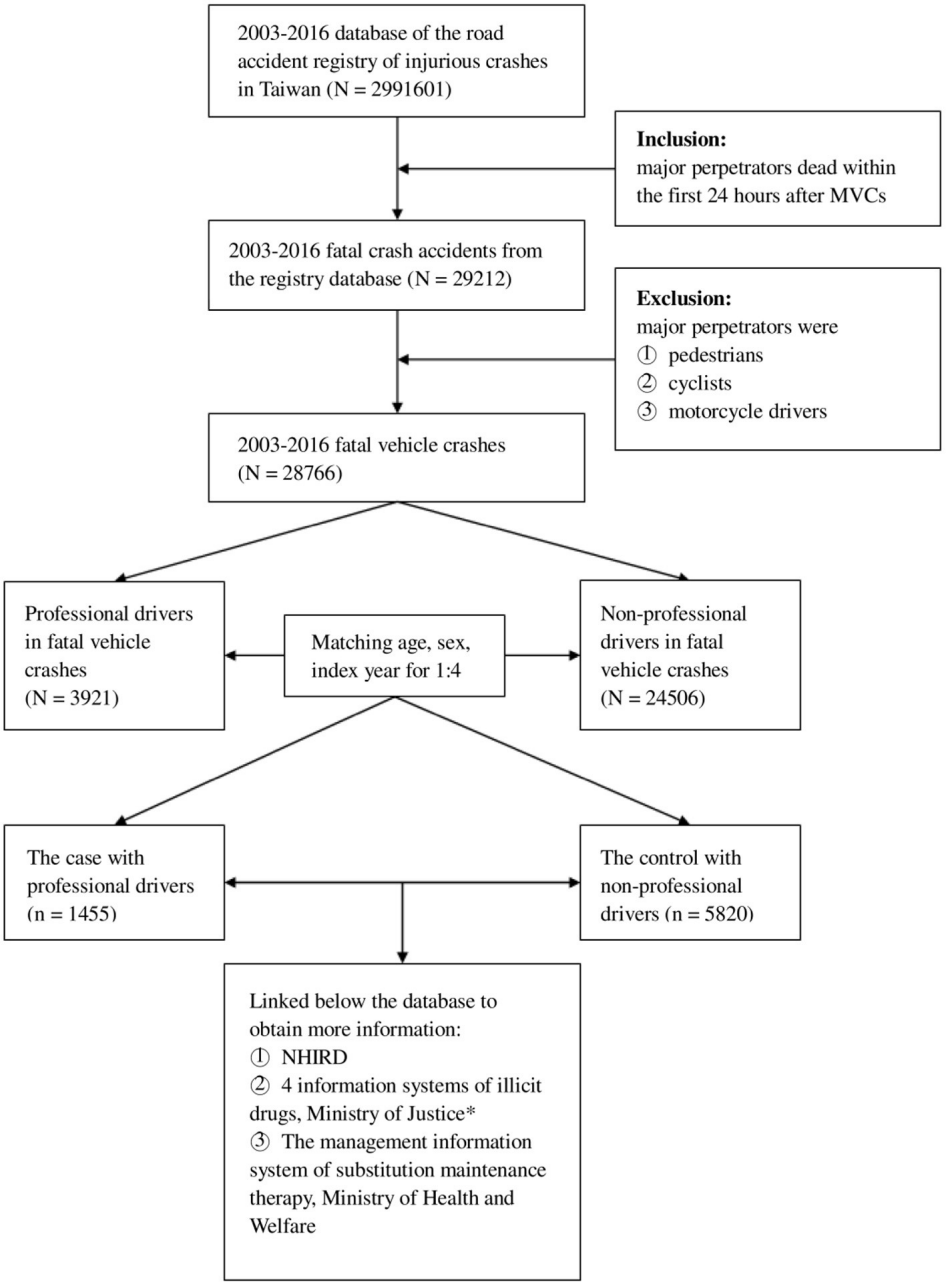
Flowchart of the descriptive study. *The four information systems were the case management system of the Drug Prevention and Control Center, criminal records processing system, criminal case system of drug case prosecutor briefed the transfer of information, and punitive administrative system for illicit drug users (category 3 or 4 narcotics). MVC, motor vehicle crash; NHIRD, Taiwan National Health Insurance Research Database.

The rate of FVCs in Taiwan from 2003 to 2016 was estimated first. FVCs were defined as vehicle crashes in which the major perpetrators died within 24 h of the incident; major perpetrators excluded pedestrians, cyclists, or motorcyclists. The preliminary study mainly used the Road Accident Registry of Injurious Crashes to identify the study population involved in FVCs from January 1, 2003, to December 31, 2016. The date (year) of the FVC was defined as the index date (year). From the FVCs, those in which the major perpetrators were professional drivers were selected as professional group. Moreover, professional drivers were defined as drivers with government-issued business driver licenses in Taiwan, including all drivers of trailer, trunk, bus, delivery, taxi, etc. To enhance the precision of the comparison, we used frequency-matched to select the comparing non-professional driver. Those with non-professional drivers were randomly selected as non-professional group after frequency-matching with the professional group at a ratio of 1:4 according to age, sex, and the index year. The flowchart of the descriptive study is depicted in Figure 1. In addition, potential covariates, including history of alcoholism, cardiovascular diseases, benzodiazepine use, involvement in motor vehicle crashes, and illicit drug abuse, as well as physical condition (Charlson comorbidity index score [CCI score]) before the index date, were included in the analysis (Supplementary Table 1) (6, 11, 12, 17–22).

### Comparing Related Factors Between Professional and Non-professional Drivers for FVCs

The data concerning conditions surrounding FVCs (collected in instances when the police immediately arrived at the scene and recorded the conditions after the FVC) were mainly retrieved from the Road Accident Registry of Injurious Crashes. The registry in-formation includes the demographic characteristics of involved parties, weather conditions, lighting conditions, car details, road conditions, and driving conditions (7, 11–13). A previous history of involvement in motor vehicle crashes was defined as the major perpetrators having had a motor vehicle crash prior to the FVC, as determined by the data collected from the Road Accident Registry of Injurious Crashes. A previous history of benzodiazepine use was defined as such use for at least 1 month within 1 year before the FVC as indicated in the NHIRD (Supplementary Table 1). In our study, we try to compare the related characteristics and to estimate interactions between professional/non-professional driver and these factors.

### Statistical Analysis

We used the chi-square test to compare the distributions of sociodemographic characteristics and potential confounding factors between the professionals and non-professionals. Logistic regression models were used to analyze the effects of single and multiple covariates on the comparison of the risk of FVCs among professional drivers. All statistical analyses were performed using SAS (version 9.3, SAS Institute, Cary, NC). A two-tailed *p* value of < 0.05 was considered statistically significant.

## Results

### Crude Incidence Rates of FVCs

**Figure 2:**
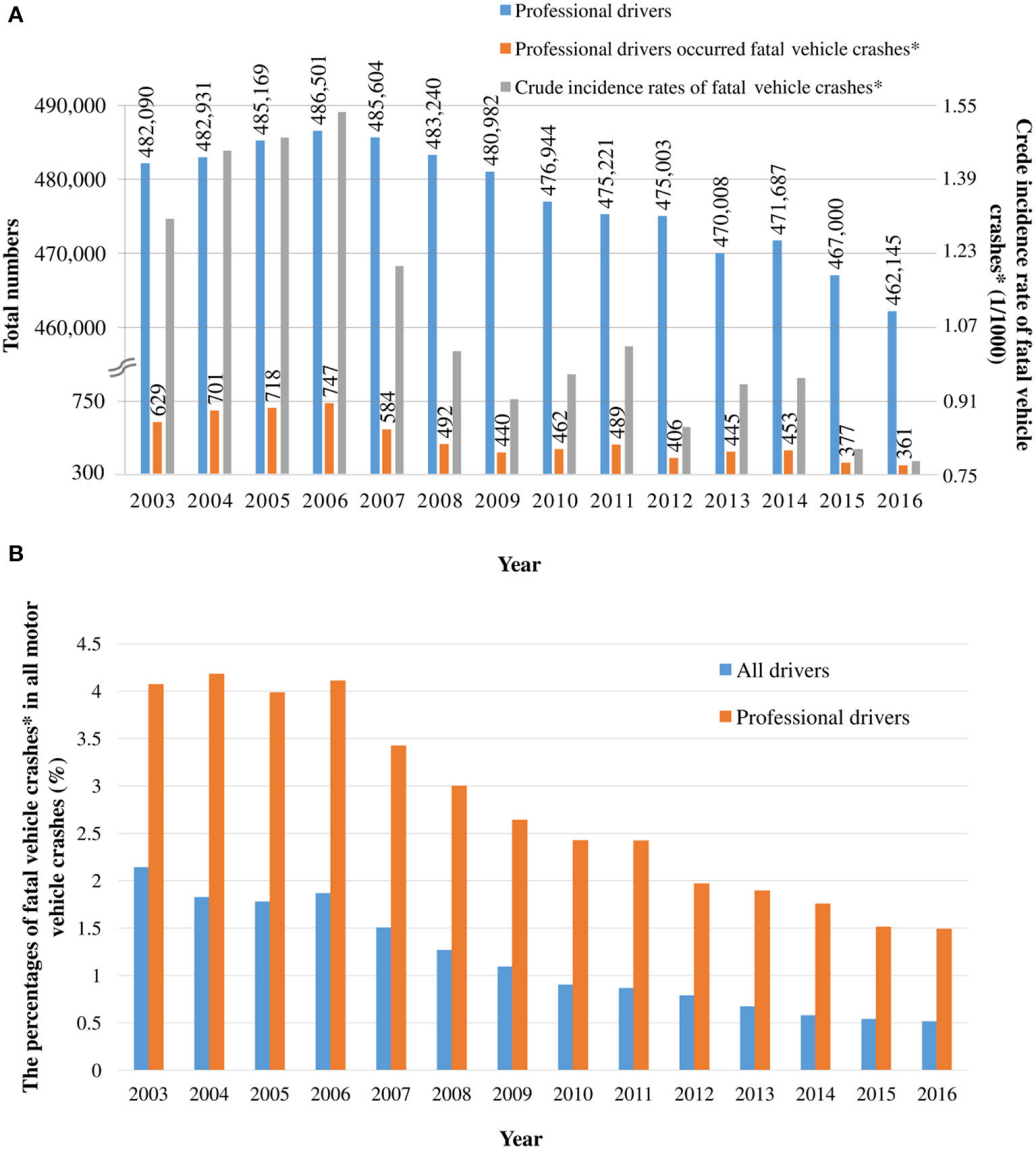
**(A)** Crude incidence rates of fatal vehicle crashes among professional drivers from 2003 to 2016. **(B)** Percentage of fatal vehicle crashes among all motor vehicle crashes from 2003 and 2016. *Fatal vehicle crashes: major perpetrators died within the first 24 h after the accident.

Figure 2 displays the crude incidence rates of FVCs in Taiwan between 2003 and 2016. The crude incidence rates of FVCs among professional drivers slightly increased from 1.30 per 1,000 person-years in 2003 to a peak of 1.53 per 1,000 person-years in 2004 and then substantially decreased to 0.78 per 1,000 person-years in 2016 (Figure 2A). Over the 14-year follow-up period, the average crude incidence rate of FVCs among professional drivers was 1.09 per 1,000 person-years; decreasingly, the average crude incidence rate of FVCs among non-professional drivers was 0.16 per 1,000 person-years. Moreover, for professional drivers, the percentages of FVCs among all motor vehicle crashes decreased from ~4% in 2003 to 1.5% in 2016; among non-professional drivers, a similar trend was observed, with a decrease from ~2–0.5% (Figure 2B). During the follow-up period, the percentages of FVCs was higher for professional drivers than for non-professional drivers. The decrease in the percentage of FVCs for professionals was approximately 1.5 times less than that for non-professional drivers.

### Study Population Characteristics

After excluding drivers who did not meet the study criteria, 28,766 drivers involved in FVCs between 2003 and 2016 were selected. Among them, 3,921 professional drivers in FVCs were enrolled as the professional group. We eventually matched 1,455 professionals to 5,820 non-professionals who were randomly selected after being frequency-matched by age, sex, and index year with the professionals at a ratio of 1:4 (Figure 1). Table 1 shows the distribution of sociodemographic characteristics and potential covariates of the professionals and non-professionals. Compared with the non-professional drivers, the professionals exhibited significantly higher percentages of residing in the north of Taiwan (*p* < 0.0001); residing in urban areas (*p* < 0.0001); having a high monthly income (*p* < 0.0001); history of benzodiazepine use (74.50 vs. 68.00%, *p* < 0.0001); and history of involvement in motor vehicle crashes (33.20 vs. 18.42%, *p* < 0.0001); however, significantly lower percentages of professionals had a history of alcoholism (3.16 vs. 6.00%, *p* < 0.0001) and history of illicit drug abuse (4.74 vs. 7.41%, *p* = 0.0003). For the other potential covariates, differences between both groups were non-significant.

**Table 1:** Baseline characteristics of the professional and non-professional groups.

	**The professionals** **(*****n*** **=** **1,455)**		**The Non-professionals** **(*****n*** **=** **5,820)**		
**Characteristics**	***n***	**(%)**	***n***	**(%)**	***p***
**Sociodemographic characteristics**					
Age, by the year 2016	45.73 ± 12.41		45.73 ± 12.41		1.000
**Sex**					
Male	1,429	98.21	5,716	98.21	1.000
Female	26	1.79	104	1.79	
Geographic region**					
Northern	537	36.91	1,776	30.52	<0.0001*
Central	375	25.77	1,763	30.29	
Southern	478	32.85	1,926	33.09	
Eastern	55	3.78	315	5.41	
Urbanization level**					
Urban	780	53.61	2,425	41.67	<0.0001*
Suburban	59	4.05	347	5.96	
Rural	606	41.65	3,008	51.68	
**Monthly income (NT$)**					
High (>30,000)	358	24.60	1,078	18.52	<0.0001*
Low (<30,000)	1,097	75.40	4,742	81.48	
**Potential covariates**					
Previous history of alcoholism	46	3.16	349	6.00	<0.0001*
CVD	12	0.80	75	1.29	0.1454
BZD use	1,084	74.5	3,957	68.0	<0.0001*
Involvement in MVCs	483	33.2	1,072	18.42	<0.0001*
Illicit drug abuse	69	4.74	431	7.41	0.0003*
CCI score					
0	1,312	90.17	5,157	88.61	0.1393
1-2	127	8.73	566	9.73	
>2	16	1.10	97	1.67	

*BZD, benzodiazepine; CCI, Charlson comorbidity index; CVD, cardiovascular disease; MVC, motor vehicle crash; NT$, new Taiwan dollar.*

*p <0.05; **Missing data: 10 professionals and 40 non-professionals.

### Study Outcome and Potential Covariates

Table 2 presents the risk factors for FVCs among professional drivers. After adjusting for geographic region, urbanization, monthly income, history, CCI score, and other fatal driving under the conditions, history of benzodiazepine use, history of involvement in motor vehicle crashes, and speeding were the only three factors that significantly affected FVCs among professional drivers, with adjusted odds ratio (OR) of 1.385 (95% confidence interval [CI], 1.215–1.579), 2.157 (95% CI, 1.896–2.453), and 1.009 (95% CI, 1.006–1.013), respectively. Among professional drivers, the following factors had significant negative associations with FVCs: history of alcoholism (adjusted OR = 0.543; 95% CI, 0.396–0.743), history of illicit drug abuse (adjusted OR = 0.646; 95% CI, 0.496–0.840), driving under the influence of alcohol (adjusted OR = 0.543; 95% CI, 0.396–0.743), driving without a seatbelt (adjusted OR = 0.543; 95% CI, 0.396–0.743), driving while distraction (adjusted OR = 0.756; 95% CI, 0.648–0.883), and driving under poor lighting conditions (adjusted OR = 0.543; 95% CI, 0.396–0.743).

**Table 2:** Risk factors for fatal vehicle crashes involving professional drivers.

**Variables**	**Crude OR (95% CI)**	***p***	**Adjusted OR^**a**^ (95% CI)**	***P***
Previous history of alcoholism	0.512 (0.374–0.700)	<0.0001*	0.543 (0.396–0.743)	0.0001*
CVD	0.638 (0.346–1.176)	0.1496	0.644 (0.347–1.192)	0.1613*
BZD use	1.376 (1.208–1.567)	<0.0001*	1.385 (1.215–1.579)	<0.0001*
Involvement in MVCs	2.201 (1.937–2.501)	<0.0001*	2.157 (1.896–2.453)	<0.0001*
Illicit drug abuse	0.622 (0.480–0.808)	0.0004*	0.646 (0.496–0.84)	0.0011*
**Fatal driving under the condition**				
Speeding	1.008 (1.005–1.012)	<0.0001*	1.009 (1.006–1.013)	<0.0001*
Alcohol use	0.240 (0.202–0.285)	<0.0001*	0.248 (0.208–0.294)	<0.0001*
Not wearing seatbelts	0.486 (0.424–0.557)	<0.0001*	0.530 (0.461–0.608)	<0.0001*
Fatigue	1.144 (0.601–2.179)	0.6822	1.164 (0.608–2.228)	0.6464
Distraction	0.744 (0.638–0.867)	0.0002*	0.756 (0.648–0.883)	0.0004*
Poor weather	1.030 (0.899–1.180)	0.6698	1.013 (0.883–1.161)	0.8583
Poor light	0.421 (0.344–0.516)	<0.0001*	0.518 (0.460–0.537)	<0.0001*
Apparent distance^b^	1.385 (1.114–1.722)	0.0033*	1.239 (0.994–1.544)	0.0567

*BZD, benzodiazepine; CCI, Charlson comorbidity index; CI, confidence interval; CVD, cardiovascular disease; MVC, motor vehicle crash; OR, odds ratio.*

*^a^Adjusted for geographic region, urbanization level, monthly income, past history, CCI score, and other fatal driving under the conditions.*

*^b^Missing data: 10 professionals and 40 non-professionals.*

*p <0.05.

## Discussion

A higher percentage of motor vehicle crashes were FVCs for the professionals than for the non-professionals; additionally, the average crude incidence rate of FVCs among professional drivers from 2003 to 2016 was 1.09 per 1000 person-years. In the 14-year preliminary study with frequency-matched non-professionals, risk factors for FVCs among professional drivers were history of involvement in motor vehicle crashes, history of benzodiazepine use, and speeding.

In the preliminary analysis based on the nationwide crude FVC incidence rates, the crude incidence rates of FVCs among professional drivers slightly increased to a peak between 2003 and 2006, which was followed by a significant decline from 2016. The decreasing tend is similar to that discussed in a previous study that attributed the trend in professional driver fatality rates in the United States from 2003 to 2016 to cutting-edge technological improvements, progressive policies, and improvements connectivity and comfort for professional drivers (7). Similar reasoning can explain the trend we observed in this study. We determined that the percentages of FVCs among professional drivers were higher than those among non-professional drivers that the decreasing trend for non-professional drivers was approximately 1.5 times higher than that for professionals. It is worth further exploring potential risk factors for FVCs among professional drivers to determine more effective measures for preventing the FVCs.

In the preliminary study with 1,455 professional drivers and 5,820 non-professional drivers, the percentage of non-professional drivers with a history of alcoholism was approximately twice that of professionals (3.16 vs. 6.00%). Similarly, the rate of history of illicit abuse was ~1.6-fold higher among non-professional drivers than among professionals (7.41 vs. 4.74%). This suggests that professional drivers in Taiwan have typically been screened and excluded for having bad habits (alcohol and substance use) before becoming professional drivers. However, the professionals were more likely to have used benzodiazepine and been in motor vehicle crashes before their index date than the non-professionals were. This suggests that the professional drivers may take benzodiazepines to endure their high job stress that perceived stress could increase risky behaviors (23) and to cope with sleep disturbance as well as indicating that their occupation is inherently dangerous.

Notably, a history of involvement in motor vehicle crashes was a major risk factor for FVCs among professional drivers in the present study. A study in Finland demonstrated that, among truck drivers, a history of involvement in a road crash during the year before the study did not have a significant effect on FVC occurrence (11). Hence, most relevant studies have suggested that driving style (inferred from histories of crashes and traffic offenses) may be a strong indicator of a driver's safety and responsibility, as evidenced in traffic offenses, motor vehicle crashes, or FVCs (2, 24–26); our results confirm this. Studies have proposed a variety of reasons to explain the aforementioned results. First, drivers who have had motor vehicle crashes may develop a tendency to intentionally take risks and recalibrate their perception of risky behavior to regard it as normal, resulting in poor self-control (27). Secondary, the drivers with a previous history of motor vehicle crashes may tend to be prone to disobeying traffic rules or speeding, deeming these as acceptable risks (28). Finally, such drivers might also become aggressive so as to escape out of fears getting along with the negative consequences of involving in the risky driving behaviors (29). The relevant authorities should carefully evaluate prospective professional drivers through qualification examinations before they become professional drivers, particularly those who have had multiple motor vehicle crashes. Professional driver education plays a crucial role in minimizing risky driving behaviors among prospective professional drivers.

Fatalities resulting from driving under the influence of drugs in the United States have been gradually increasing while, since 2000, the variety of intoxicants has shifted from illegal drugs to prescription medications, especially in benzodiazepine (30). Benzodiazepines have become one of most widely detected drugs in post mortems of drivers involved in FVCs (31). A growing body of evidence confirms that the use of benzodiazepines is significantly associated with an increased risk of injurious crashes and FVCs (32–35). We could only identify a history of benzodiazepine use before an FVC from the NHIRD. Despite lacking data on benzodiazepine use during FVCs, a history of benzodiazepine use was associated with an increased risk of FVCs among professional drivers. Moreover, several forensic research studies have found that the drivers who took two or more types of benzodiazepines have a significantly higher risk of FVCs than those who took only one such prescription (31–33). Our findings were similar; 58% of drivers in FVCs had been received at least two types of benzodiazepines and 42% had been given only one such prescription (Supplementary Table 2). The relationship between FVCs and benzodiazepine use is disputed. Several experimental studies have shown that drivers using benzodiazepines perform worse on driving simulator tests compared with those receiving a placebo (36, 37). Benzodiazepines are known to produce dose-related impairment of concentration, reaction time, and psychomotor function in human beings (37), which may partially explain the correlation. Thus, authorities, policy makers, and professional drivers should be aware of this risk, and drivers should be monitored for benzodiazepine use before driving. In addition, early intervention and treatment for underlying diseases necessitating benzodiazepine use may be more beneficial than conventional punishments for drivers who use benzodiazepine.

Driving speed is also a major factor in motor vehicle crashes (38). The relationship between speeding and FVCs has been well-researched (39, 40). For example, Bédard et al. (39) indicated that driving at a speed of 113 km/h or more resulted in an ~1.64-fold higher risk of FVCs than driving at a speed of 56 km/h or lower. Our results were in agreement with this finding. For professional drivers, speeding may be perceived as a means of increased income by minimize travel time and increase trips made or goods delivered, with the result being an increased risk of FVCs. Therefore, legislation regarding speed limits and heightened law enforcement of speeding violations may reduce speeding-related fatalities. Moreover, we suggest professional drivers regularly adjusting themselves to speeding behavior, as average speech enforcement (41).

At first glance, a history of alcoholism, a history of illicit drug abuse, driving under the influence of alcohol, driving without a seatbelt, driving while distracted, and driving under poor lighting conditions were not consequential risk factors (all adjusted OR <1) for FVCs among professional drivers in our study. However, these factors have been recognized as risk factors for FVCs in all drivers (9, 11, 17–20). We compared the potential recognized risk factors for FVCs between professional and non-professional drivers in our study design. Thus, non-professional drivers had higher occurrence of FVCs underlying the afore-mentioned factors than professional drivers. That also implied that compared to non-professional derivers, professional drivers could avoid having such dangerous behaviors such as driving under the influence of alcohol with the guild demands or strict laws in our society. Overall, the factors (such as a history of alcoholism, a history of illicit drug abuse, driving under the influence of alcohol, driving without a seatbelt, driving while distracted, and driving under poor lighting conditions) still contribute to an increased risk of FVCs in both professional and non-professional populations; underlying the factors, non-professional drivers had higher risk of FVCs than professional drivers.

### Strengths and Limitations

The major strength of our study is the use of a population-based sample of nation-wide FVC incidence rates and a preliminary study design for exploring the potential risk factors for FVCs among professional drivers. The findings may provide a basis for designing measures to prevent FVCs among professional drivers, particularly in ethnic Chinese populations. Moreover, the data were not subject to reporting or recall bias because we used high-quality data from six administrative registries and the NHIRD.

Nevertheless, we acknowledge several possible limitations of this study. First, based on the limitation from our databases, we only compared the related characteristics and potential risk factors for FVC between professional and non-professional drivers. That may lead to inevitable confounding effects such as driver duration, frequency of road use, and so on. For example, involvements in motor vehicle crashes may reflect a higher frequency of road uses among professional drivers than those among non-professional drivers. FVCs and motor vehicle crashes in the study were assessed using data from a police registry. Because unreported or minor motor vehicle crashes would not have been included in our analysis, the total number of motor vehicle crashes may have been underestimated; however, FVCs were accurately documented. Second, data on fatalities when driving under the influence of drugs, such as benzodiazepines and illicit drugs, were not obtained from the Road Accident Registry of Injurious Crashes. Therefore, we recorded a history of benzodiazepine use and illicit drug abuse before an FVCs in this study. Third, information on potentially significant individual confounders, such as risk-taking behaviors and poor impulse control, was not available. Fourth, accurate information regarding risk factors for FVCs may be difficult to check because we used database information. Finally, the NHIRD only provides information regarding the dispensing of prescribed medications. Because non-adherence is considered a potential confounder, caution should be exercised when comparing our findings with the results reported by other groups in which data were collected from clinical settings (3–5).

## Conclusion

In line with previous findings, we identified compelling evidence that professional drivers have a higher risk of FVCs than do non-professional drivers and that the three major risk factors for FVCs among professional drivers are a history of involvement in motor vehicle crashes, a history of benzodiazepine use, and speeding. Relevant authorities, policy makers, and professional drivers should be aware of this risk, and appropriate measures should be implemented to prevent FVCs.

## Data Availability Statement

The original contributions presented in the study are included in the article/Supplementary Material, further inquiries can be directed to the corresponding authors.

## Ethics Statement

This study used seven nationwide databases retrieved from the Road Accident Registry of Injurious Crashes maintained by the National Police Agency, Ministry of the Interior (14, 15); the Taiwan National Health Insurance Research Database (NHIRD) (15, 16); and five Taiwanese population68 based administrative registries, namely the management information system of substitution maintenance therapy (Ministry of Health and Welfare) and four information management systems concerning illicit drugs that are maintained by the Ministry of Justice (10). The four information systems concerning illicit drugs were the case management system of the Drug Prevention and Control Center, the criminal records processing system, the drug case prosecution case system, and the punitive administration system for illicit drug users (category 3 or 4 narcotics) (Supplementary Table 3). Preliminary data were independently collected by the aforementioned governmental departments and managed by the Health and Welfare Data Science Center, Ministry of Health and Welfare. All data from the various systems were linked using the unique national identification numbers assigned to each resident in Taiwan. Personal identifiers were removed after linking and before the analysis. The study was approved by the relevant institutional review boards (TSMH IRB No.: 17-010-B1 and 18-049-B), and written informed consent was waived because the data we obtained were de-identified.

## Author Contributions

J-HT, H-YC, and Y-HY: conceptualized and led the study. J-HT and Y-HY: data curation and project administration. Y-HY and P-SH: formal analysis. J-HT, P-SH, and H-YC: methodology. T-NW, H-YC, P-CC, and YG: supervision. All authors contributed to the article and approved the submitted version.

## Funding

This study was supported by the funds provided by 4 grants from the Taiwan Food and Drug Administration (TFDA) (MOHW106-FDA-D-114-000671 and MOHW107-FDA-D-114-000636), the Research Center for Environmental Medicine, Kaohsiung Medical University, Kaohsiung, Taiwan from The Featured Areas Research Center Program within the framework of the Higher Education Sprout Project by the Ministry of Education (MOE) in Taiwan, and by Kaohsiung Medical University Research Center Grant (KMUTC109A01-1).

## Conflict of Interest

The authors declare that the research was conducted in the absence of any commercial or financial relationships that could be construed as a potential conflict of interest.

## Publisher's Note

All claims expressed in this article are solely those of the authors and do not necessarily represent those of their affiliated organizations, or those of the publisher, the editors and the reviewers. Any product that may be evaluated in this article, or claim that may be made by its manufacturer, is not guaranteed or endorsed by the publisher.
