# Supplementary material for: Incidence and Risk of Fatal Vehicle Crashes Among Professional Drivers: A Population-Based Study in Taiwan
Source: Front Public Health. 2022 Mar 8;10:849547. doi: 10.3389/fpubh.2022.849547 (PMC8957854; doi:10.3389/fpubh.2022.849547)
Supplement: Supplementary file 1 [file Table_1.DOCX]

| SUPPLEMENTAL TABLE 1｜ICD-9-CM and ATC codes used in this study. | |
| --- | --- |
| **Disease categories** | **ICD-9-CM codes** |
| Alcoholism | 291, 303.0X, 303.9, 305.00–305.03, 357.5, 425.5, 535.30, 535.31, 571.0–571.5, 571.8, 571.9, 790.3, 980.0, 980.2, 980.8, 980.9, 977.3, V11.3 |
| Cardiovascular diseases (CVD) | 410, 428, 441, 434.01, 434.11, 430, 431, 437.2 |
| **Drug categories** | **ATC codes** |
| Benzodiazepine (BZD) |  |
| Clonazepam | N03AE01 |
| Diazepam | N05BA01 |
| Chlordiazepoxide | N05BA02 |
| Medazepam | N05BA03 |
| Oxazepam | N05BA04 |
| Potassium clorazepate | N05BA05 |
| Lorazepam | N05BA06 |
| Adinazolam | N05BA07 |
| Bromazepam | N05BA08 |
| Clobazam | N05BA09 |
| Ketazolam | N05BA10 |
| Prazepam | N05BA1 |
| Alprazolam | N05BA12 |
| Halazepam | N05BA13 |
| Pinazepam | N05BA14 |
| Camazepam | N05BA15 |
| Nordazepam | N05BA16 |
| Fludiazepam | N05BA17 |
| Ethyl loflazepate | N05BA18 |
| Etizolam | N05BA19 |
| Clotizepam | N05BA21 |
| Cloxazolam | N05BA22 |
| Tofisopam | N05BA23 |
| Lorazepam, combinations | N05BA56 |
| Flurazepam | N05CD01 |
| Nitrazepam | N05CD02 |
| Flunitrazepam | N05CD03 |
| Estazolam | N05CD04 |
| Triazolam | N05CD05 |
| Lormetazepam | N05CD06 |
| Temazepam | N05CD07 |
| Midazolam | N05CD08 |
| Brotizolam | N05CD09 |
| Quazepam | N05CD10 |
| Loprazolam | N05CD11 |
| Doxefazepam | N05CD12 |
| Cinolazepam | N05CD13 |
| Zopiclone | N05CF01 |
| Zolpidem | N05CF02 |
| Zaleplon | N05CF03 |
| Eszopliclone | N05CF04 |
| *ATC, Anatomical Therapeutic Chemical; ICD-9-CM, International classification of diseases, ninth revision, clinical modification.* | |
