# Supplementary material for: Incidence and Risk of Fatal Vehicle Crashes Among Professional Drivers: A Population-Based Study in Taiwan
Source: Front Public Health. 2022 Mar 8;10:849547. doi: 10.3389/fpubh.2022.849547 (PMC8957854; doi:10.3389/fpubh.2022.849547)
Supplement: Supplementary file 2 [file Table_2.DOCX]

| SUPPLEMENTAL TABLE 2｜The type(s) of benzodiazepine use among professional drivers in this study. | | |
| --- | --- | --- |
| **The type(s) of BZD use** | **n** |  |
| 1 | 453 |  |
| 2 | 279 |  |
| 3 | 139 |  |
| 4 | 97 |  |
| 5 | 51 |  |
| 6 | 28 |  |
| 7 | 15 |  |
| 8 | 9 |  |
| 9 | 3 |  |
| 10 | 4 |  |
| More than 10 | 6 |  |
| *BZD, benzodiazepine.* | |  |
