# Supplementary material for: Incidence and Risk of Fatal Vehicle Crashes Among Professional Drivers: A Population-Based Study in Taiwan
Source: Front Public Health. 2022 Mar 8;10:849547. doi: 10.3389/fpubh.2022.849547 (PMC8957854; doi:10.3389/fpubh.2022.849547)
Supplement: Supplementary file 3 [file Table_3.DOCX]

| SUPPLEMENTAL TABLE 3｜The data of each variable in the study were derived from these datasets. | |
| --- | --- |
| **Characteristics & variable** | **Derived datasets** |
| Professional drivers in FVAs | RARIC |
| Nonprofessional drivers in FVAs | RARIC |
| Sociodemographic characteristics |  |
| Age | NHIRD, RARIC |
| Sex | NHIRD, RARIC |
| Geographic region | NHIRD |
| Urbanization level | NHIRD |
| Monthly income (NT$) | NHIRD |
| Previous history of |  |
| Alcoholism | NHIRD |
| CVD | NHIRD |
| BZD use | NHIRD |
| Involvement in MVAs | RARIC |
| Illicit drug abuse | MISSSMT, 4IMISID |
| CCI score | NHIRD |
| Fatal driving under the condition | RARIC |
| *BZD, benzodiazepine; CCI, Charlson comorbidity index; CVD, cardiovascular disease; FVAs, fatal vehicle accidents; MISSSMT, Management Information System of Substitution Maintenance Therapy, Ministry of Health and Welfare; MVA, motor vehicle accident; NHIRD, Taiwan National Health Insurance Research Database; NT$, new Taiwan dollars; RARIC, Road Accident Registry of Injurious Crashes, National Police Agency, Ministry of the Interior; 4IMISID, 4 independent management information systems of illicit drugs, Ministry of Justice, which composed of the case management system of Drug Prevention and Control Center, processing system of criminal records, criminal case system of drug case prosecutor briefed the transfer of information, and punitive administrative system for the use of Category 3 or 4 Narcotics (illicit drugs).* | |
